# CaMKII as a Multimodal Signalling Hub in Neural Connectivity and Vulnerability

**DOI:** 10.1007/s12035-026-05777-0

**Published:** 2026-03-18

**Authors:** Stephanie Olliff, Vinod Sundaramoorthy

**Affiliations:** 1 Commonwealth Scientific and Industrial Research Organisation (CSIRO), Australian Centre for Disease Preparedness (ACDP), East Geelong, VIC Australia; 2 School of Medicine, Deakin University, Geelong, VIC Australia

**Keywords:** CaMKII signalling, Synaptic plasticity, Axon growth and degeneration, Kinase–phosphatase balance, Protein phosphatase, CaMKII isoform diversity

## Abstract

Calcium/calmodulin-dependent protein kinase II (CaMKII) is one of the most abundant and versatile signalling molecules in the brain, uniquely positioned to convert transient signals into durable structural and functional changes. Classical models cast CaMKII as a Ca^2+^/calmodulin-activated kinase that, once phosphorylated, persists autonomously to encode synaptic memory. Recent work has reframed this view, revealing CaMKII as a state and context-dependent signalling hub that integrates catalytic activity with structural and scaffolding functions. Its activity and persistence are shaped by partner protein interactions, higher-order assembly, redox and metabolic modifications, and confinement within nanoscale domains. Isoform and splice diversity further distribute CaMKII into specialised pools across dendritic spines, growth cones, axons, and nuclei, enabling it to regulate synaptic plasticity, axon growth, and long-term neuronal stability through both enzymatic and non-enzymatic mechanisms. These actions are dynamically sculpted by opposing phosphatases, including PP1, PP2A, and calcineurin, which do not simply terminate signalling but bias the kinase toward kinetically stabilised functional configurations with distinct catalytic and structural outputs. Here we outline a conceptual multistate regulatory framework in which CaMKII can occupy basal, catalytically active, and structurally stabilised functional regimes, rather than operating as a simple bistable switch. In this framework, these regimes are defined by differences in signalling persistence and molecular interactions rather than by formally demonstrated stable attractors, providing a functional model for understanding how CaMKII integrates spatially restricted Ca^2+^ signals with phosphatase control. Disruption of regulated transitions between these regimes through altered phosphatase balance, aberrant spatial confinement, or pathological modification can shift CaMKII signalling into inappropriate compartments or timescales, contributing to neurodevelopmental, psychiatric, and neurodegenerative disorders. This review highlights recent advances in activation mechanisms, isoform-specific organisation, phosphatase control, and disease biology, positioning CaMKII as a hybrid structural–catalytic integrator whose state-dependent regulation offers new opportunities for precision therapeutic intervention.

## Introduction

Calcium/calmodulin-dependent protein kinase II (CaMKII) comprises 1–2% of total brain protein and is one of the most powerful molecular switches in the nervous system [1]. It has the unique ability to transform transient calcium signals into long-lasting changes in neuronal structure and function [2]. The CaMKII holoenzyme, assembled as a dodecameric complex from four isoforms (α, β, γ, and δ), is structurally designed to integrate and sustain signalling [3]. Mixed α/β holoenzymes predominate in forebrain neurons and provide cooperative activation, inter-subunit autophosphorylation, and the capacity for subunit exchange between holoenzymes [4]. Subunit exchange has been demonstrated most clearly for CaMKIIα/β holoenzymes, while structural and biochemical analyses suggest that related hub architectures in other isoforms could support similar exchange under appropriate activation conditions [5–7].

**Fig. 1:**
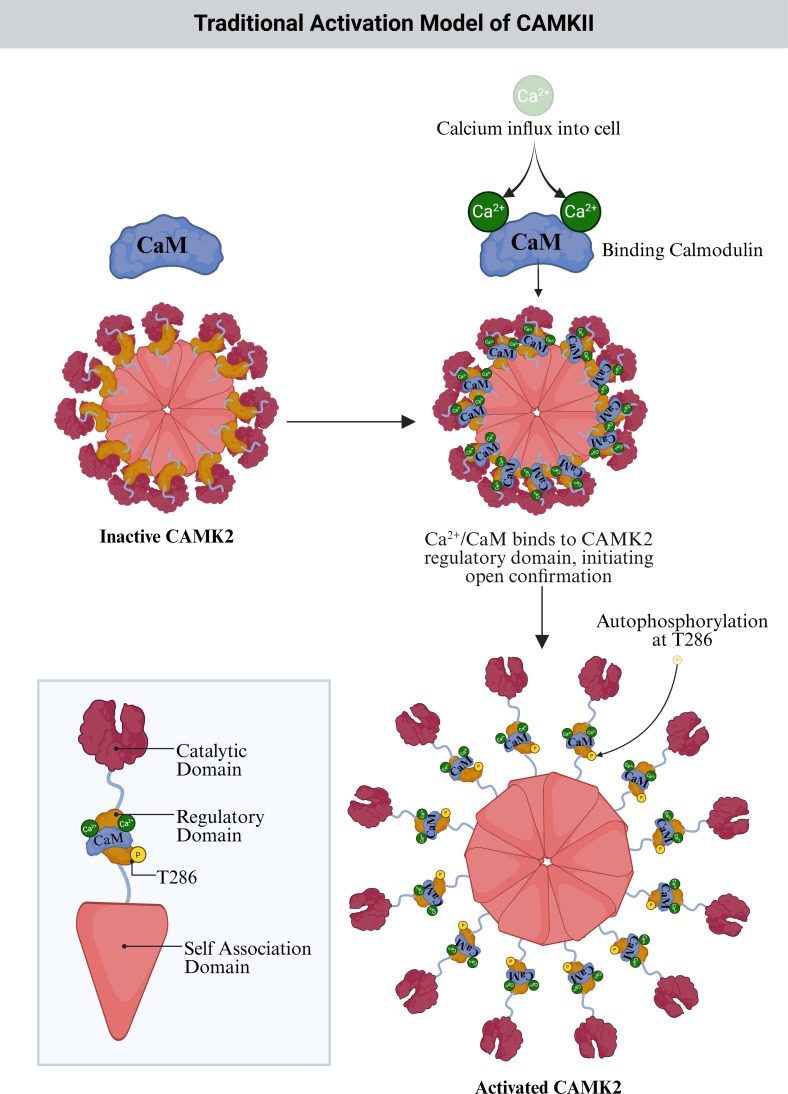
Traditional activation model of CaMKII by Ca^2+^/calmodulin. Calcium influx enables Ca^2+^/calmodulin (CaM) binding to the regulatory domains of inactive CaMKII, displacing autoinhibition and exposing the catalytic sites. Subsequent autophosphorylation at threonine 286 (T286) stabilises the active conformation. Inset shows a single subunit with catalytic, regulatory, and self-association domains. *Made with BioRender*

Traditionally, CAMKII has been framed as a Ca^2+^/calmodulin (CaM)-activated kinase that autophosphorylates at Thr286/Thr287 and then persists in an autonomous state to encode synaptic memory and learning (Fig. 1) [8]. This model has shaped decades of work on synaptic plasticity and memory formation, but an increasing body of structural and biochemical data now shows that it captures only part of CaMKII biology. The kinase operates within a broader and more complex regulatory landscape in which partner binding, higher-order assembly, phosphatase feedback, and isoform-specific localisation collectively determine when, where, and how CaMKII remains active [2, 9, 10].

Binding to partner proteins, regulatory segment mediated dimerisation, activation-triggered subunit exchange, confinement within phase-separated nanodomains, and redox or metabolite driven post-translational modifications can each stabilise Ca^2+^ independent sustained activity [9, 11–17]. Together, these modes position CaMKII not as a simple Ca^2+^ gated enzyme but as a context-dependent signalling hub whose persistence can be structural, catalytic, or both.

This mechanistic flexibility is tightly coupled to CaMKII’s spatial and isoform diversity. All four isoforms are expressed in the brain, but CaMKIIα and CaMKIIβ dominate in excitatory neurons and are heavily enriched at postsynaptic densities where they support LTP and structural spine plasticity [18–23]. CaMKIIγ and CaMKIIδ, by contrast, show broader expression patterns and participate in nuclear signalling, calcium handling, and plasticity in both central and peripheral tissues [24]. Extensive alternative splicing, particularly within the variable linker region, generates dozens of splice variants with distinct localisation, Ca^2+^/calmodulin sensitivity, actin-binding capacity, and autophosphorylation kinetics [10, 25–28]. These isoforms and splice-specific properties underlie the very different behaviours of CaMKII in dendritic spines, growth cones, axons, and the nucleus and are central to understanding how a single kinase family can coordinate synaptic plasticity, axon growth, and long-term neuronal stability.

Within neurons, CaMKII activity is organised into nanoscale compartments that couple local calcium influx to specific structural and transcriptional outcomes [28–30]. At synapses, CaMKII shapes both neurotransmitter release and postsynaptic receptor function, working together with core scaffolding proteins to tune excitability and trafficking [31]. These local actions are complemented by CaMKII-driven phosphorylation of transcriptional regulators and cytoskeletal components, which couple synaptic activity to broader programmes of gene expression and dendritic remodelling [32]. In parallel, CaMKII in axons and growth cones interprets calcium dynamics to guide motility, maintain cytoskeletal architecture, regulate bouton formation, and support axonal resilience [33]. Together, these distributed signalling roles allow CaMKII to influence neuronal communication, structure, and survival across multiple compartments [34, 35].

Such persistent and compartmentalised activity requires equally sophisticated regulation. This nuanced control relies heavily on the coordinated actions of the major neuronal phosphatases PP1, PP2A, and calcineurin. These phosphatases do not merely terminate CaMKII signalling or selectively promote LTD; rather, they shape a regulatory landscape that biases CaMKII toward kinetically stabilised functional configurations with distinct catalytic and structural outputs [2, 10, 31, 36].

Classical models described CaMKII-phosphatase interactions as a bistable toggle between low and high phosphorylation states. However, accumulating biochemical, structural, and cell biological observations support a broader multistate regulatory framework in which CaMKII can occupy a basal configuration, a catalytically engaged LTP-like regime, and a structurally stabilised configuration in which CaMKII remains spatially retained within PSD scaffolds or biomolecular assemblies even as catalytic output diminishes [37–39]. Whether these regimes constitute formal dynamical “tristability” remains unresolved. Instead, this framework provides a functional model for integrating phosphatase feedback, spatial confinement, and non-canonical persistence mechanisms that extend CaMKII signalling beyond transient Ca^2+^ elevations.

Phosphatase-driven regulation of CaMKII is not restricted to synapses. Similar coordinated control by PP1, PP2A, and calcineurin operates in growth cones, where CaMKII activity is tuned to regulate cytoskeletal dynamics and axon guidance responses [40]. Disruption of kinase or phosphatase activity, localisation, or feedback control is increasingly linked to neurodevelopmental, psychiatric, and neurodegenerative disorders [41–46].

Therapeutically, CaMKII is therefore both an appealing and a challenging target. Isoform redundancy, extensive alternative splicing, and tightly regulated spatiotemporal activation make it difficult to modulate CaMKII without compromising essential plasticity and survival functions [7, 47, 48]. At the same time, these features open the possibility of more selective interventions that target particular isoforms, subcellular pools, or regulatory mechanisms such as structural GluN2B binding, redox-sensitive modifications, or specific phosphatase interactions. Although such precision targeting is inherently more difficult to achieve than global kinase inhibition, it offers a rational path to reshape pathological CaMKII signalling while preserving its indispensable physiological roles.

In this review, we treat CaMKII as a hybrid structural and catalytic switch rather than a simple CaM trapping memory molecule. We first revisit the classical model of Ca^2+^/calmodulin driven activation and Thr286-dependent persistence and then examine alternative modes of modulation including partner binding, higher order structural dynamics, redox and metabolic regulation, and phase-separated nanodomains. We next consider how isoform-specific localisation in spines, growth cones, axons, and nuclei shapes CaMKII function, and highlight key kinase-dependent substrates that couple CaMKII activity to synapse formation, structural plasticity, transcriptional control and axonal growth. Finally, we discuss how phosphatases contribute to the organisation of CaMKII into multistate structural and catalytic conformations across synaptic and developmental contexts. We also discuss how CaMKII isoforms influence axon growth, maintenance, and susceptibility to degeneration and how these insights can be leveraged in the search for targeted therapeutic strategies.

## CaMKII Activation and Persistence

### Activation by Calcium Calmodulin (Ca^2+^/CaM) and Autophosphorylation

The traditional model of CaMKII activation centres on a transient rise in intracellular calcium following synaptic or electrical activity [49–52]. In this long-standing framework, calcium ions bind cooperatively to calmodulin, inducing a structural rearrangement that exposes hydrophobic surfaces capable of engaging the calmodulin binding region within CaMKII’s regulatory domain (Fig. 1) [49, 53, 54]. This interaction displaces the autoinhibitory segment, which normally blocks the catalytic site through a pseudosubstrate sequence, thereby unlocking kinase activity [55–57]. Phosphorylation further stabilises this active conformation and markedly increases the enzyme’s affinity for calmodulin, a property known as calmodulin trapping, which increases CaM affinity by up to 1000- to 10,000-fold and slows its dissociation [50, 58, 59]. Importantly, both basal and phosphorylation-enhanced CaM affinity differ between CaMKII isoforms, with non‑α isoforms (such as β, and in some contexts γ and δ) generally exhibiting distinct CaM-binding kinetics and sensitivities that tune their activation thresholds and temporal integration of calcium signals relative to α [24, 60]. Functionally, CaM trapping was thought to prolong CaMKII activity well beyond the initial calcium transient, supporting sustained substrate phosphorylation required for long-term potentiation (LTP) and synaptic strengthening [2]. The classical model proposed that autophosphorylation at Thr286, or Thr287 in non-alpha isoforms, generated a Ca^2+^-independent autonomous form of CaMKII (Fig. 1). Once one subunit within the dodecameric holoenzyme became autonomous, it was believed to trigger Thr286 phosphorylation on neighbouring subunits, creating a self-perpetuating state that resisted phosphatase activity and stabilised synaptic change [61]. The trapped CaM was also thought to sterically restrict access of PP1, further stabilising the active state [62].

Modelling and structural studies supported this view by showing slowed deactivation kinetics within spines, CaM tightly wrapping the CaM binding domain, and subunit cross-activation enabled by flexible linker regions [3, 10, 62]. Although this framework (Fig. 1) shaped decades of understanding, recent evidence shows that CaMKII activation is more nuanced than CaM trapping or Thr286/Thr287 autophosphorylation alone can explain [62]. Autonomous activity and its spread within holoenzymes appear more constrained and context-dependent than originally assumed, with isoform-specific organisation, partner-driven nanoscale compartmentalisation, and local signalling environments jointly determining where and for how long CaMKII remains active [2, 4, 19–24, 27, 28, 30, 63–78]. In this framework, interactions with synaptic and non-synaptic binding partners not only influence kinase activation state but also govern its spatial confinement, integrating local protein networks and molecular architecture into higher-order control of CaMKII signalling. These insights motivate a reframing of CaMKII regulation, highlighting additional regulatory pathways and organisational mechanisms that extend well beyond the classical model of activation and persistence.

### Alternative Modes of CaMKII Modulation Beyond Kinase Catalysis

Emerging evidence has refined the classical model in which Ca^2+^/calmodulin binding and Thr286 autophosphorylation were thought to generate a Ca^2+^-independent autonomous state that maintained LTP and memory [70, 79–81]. Recent work suggests that this mechanism alone is insufficient to explain the persistence, spatial confinement, and functional diversity of CaMKII signalling observed during synaptic plasticity and neuronal development [52, 62, 72], indicating the need for additional regulatory layers that integrate catalytic activity with structural interactions, phosphatase feedback, and compartment-specific organisation. In hippocampal slices from Thr286Ala knock-in mice, an ATP-competitive CaMKII inhibitor unexpectedly rescued LTP by enhancing regulated CaMKII binding to GluN2B [82]. These findings demonstrate that, under specific experimental conditions, LTP induction can occur with minimal CaMKII catalytic output, highlighting an essential structural and scaffolding contribution of CaMKII during the induction phase. Importantly, this observation does not negate the requirement for CaMKII kinase activity in downstream consolidation, as catalytic phosphorylation remains critical for AMPAR regulation, cytoskeletal remodelling, and transcriptional programmes that stabilise plasticity [16, 83–85]. Rather than overturning the canonical model, this data indicates that Thr286 phosphorylation and kinase activity gate access to additional non-catalytic signalling modes including GluN2B anchoring and nanoscale retention which cooperate with enzymatic signalling to shape synaptic outcomes [70, 82].

#### Sustained Activation Through Protein Interactions

**Fig. 2:**
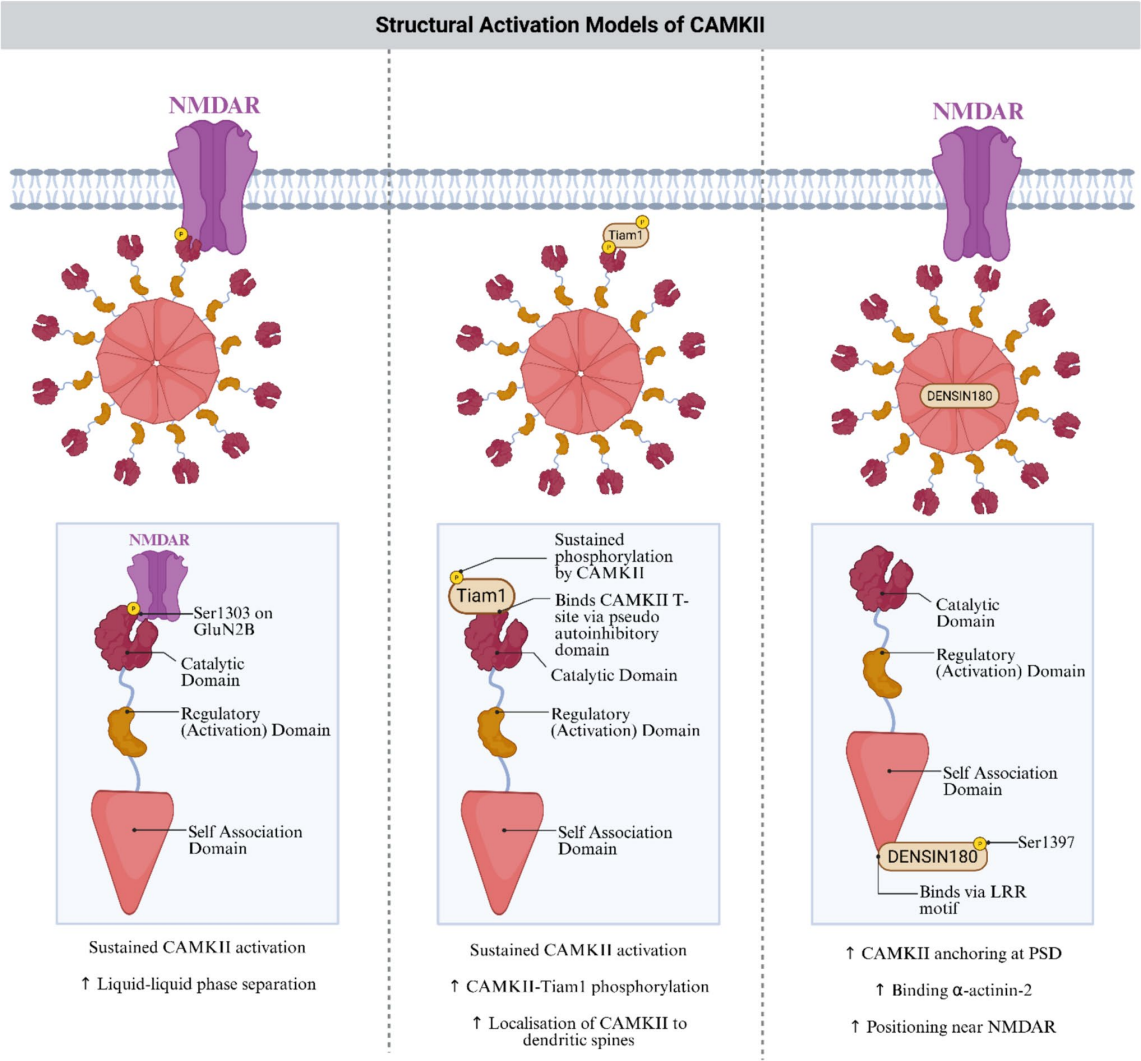
Sustained CaMKII activation through protein–protein interactions. CaMKII activation is prolonged and spatially stabilised by diverse protein–protein interactions that operate at the postsynaptic membrane. Left: Binding of CaMKII to the GluN2B subunit of the NMDA receptor maintains CaMKII in an activated conformation following Ca^2+^/CaM dissociation, supporting persistent kinase activity. This interaction also has been shown to support LLPS-like clustering in reconstitution/overexpression systems of CaMKII-GluN2B complexes, suggesting a multivalent clustering mechanism that can enrich CaMKII locally and extend its signalling lifetime. Centre: The Rac1 GEF Tiam1 binds the CaMKII T-site through a pseudo-autoinhibitory motif and is phosphorylated by CaMKII, creating a reciprocal interaction that sustains kinase activation and facilitates CaMKII accumulation within dendritic spines. Right: The scaffold protein densin-180 anchors CaMKII at the postsynaptic density (PSD) through its LRR motif and coordinates interactions with α-actinin-2, positioning CaMKII near NMDA receptors and supporting localised, prolonged activity. Together, these molecular partners stabilise active CaMKII populations, shape synaptic nanodomains, and reinforce downstream structural and signalling responses. *Made with BioRender*

Several non-canonical activation modes rely on protein interactions that stabilise an open conformation or retain CaMKII at specific subcellular sites (Fig. 2). One well characterised mechanism is CaMKII binding to the GluN2B subunit of NMDA receptors, which can maintain CaMKII in an active structural state long after Ca^2+^ signals decay and can thereby sustain synaptic potentiation [30]. Either Thr286 phosphorylation or GluN2B binding can keep the regulatory domain displaced even after Ca^2+^/calmodulin has dissociated, allowing continued Ca^2+^-independent autonomous activity, although this activity remains lower than maximal Ca^2+^/calmodulin stimulated activity [14, 70, 86–89]. This interaction has also been proposed to involve LLPS-like clustering driven by multivalent, low-affinity interactions that can locally concentrate CaMKII and GluN2B within synaptic nanodomains. To date, most evidence for CaMKII-driven phase separation derives from in vitro reconstitution systems or models involving CaMKII overexpression, which enable mechanistic dissection but may exaggerate condensate formation [66, 72, 90]. Whether comparable assemblies form at endogenous CaMKII expression levels under basal physiological conditions remains unresolved. Nevertheless, these studies establish the mechanistic principle that multivalent interaction–driven clustering can organise CaMKII into spatially restricted signalling assemblies, likely contributing to nanoscale CaMKII organisation even in the absence of large, stable condensates. Such local concentration mechanisms may help explain how CaMKII remains spatially retained and structurally engaged without continuous catalytic turnover.

CaMKII also interacts with the Ser831 region of the AMPA receptor subunit GluA1, contributing to receptor trafficking and synaptic scaling [71]. However, current evidence does not support the idea that interaction/phosphorylation at Ser831 independently drives CaMKII autonomous activity in the same manner as GluN2B binding. Instead, GluA1-associated modulation appears to operate within a broader network of protein interactions, including phase-separated assemblies, that shape CaMKII retention and local signalling [72–74].

Other interaction partners, including Tiam1 and densin 180, also influence CaMKII localisation and structural activation [75–78]. Many additional synaptic and cytoskeletal proteins are likely to contribute but remain to be fully identified. Together, these mechanisms illustrate that CaMKII activity duration, subcellular positioning, and signalling specificity can be maintained by structural interactions and condensate formation rather than by canonical Thr286 autophosphorylation.

#### Dimerisation and Activation Triggered Subunit Exchange

CaMKII structural plasticity provides another layer of regulation. Electron microscopy and integrative modelling show that a subset of CaMKIIα kinase domains form regulatory segment mediated dimers [91]. These dimers stabilise a primed intermediate state and lower the Ca^2+^/CaM threshold for cooperative activation [92]. Upon activation, CaMKII undergoes a dynamic process of subunit exchange. Ca^2+^/CaM binding together with phosphorylation at Thr286 loosens the interactions between the hub domain and the regulatory segments, reducing the structural cohesion of the holoenzyme [5]. As a result, activated kinase domains, including those arranged as transient dimers, can disengage from their original holoenzyme and incorporate into neighbouring complexes. This exchange mechanism allows activated subunits to spread their conformational state across a broader population of holoenzymes, extending the spatial range and lifetime of CaMKII signalling [93]. This creates a structural amplification system in which local activation spreads across holoenzymes, supporting prolonged synaptic signalling and allowing CaMKII to integrate transient Ca^2+^ bursts into long-lasting synaptic changes [6, 91].

#### Cytoskeletal and Scaffold-Mediated Retention Mechanisms

**Fig. 3:**
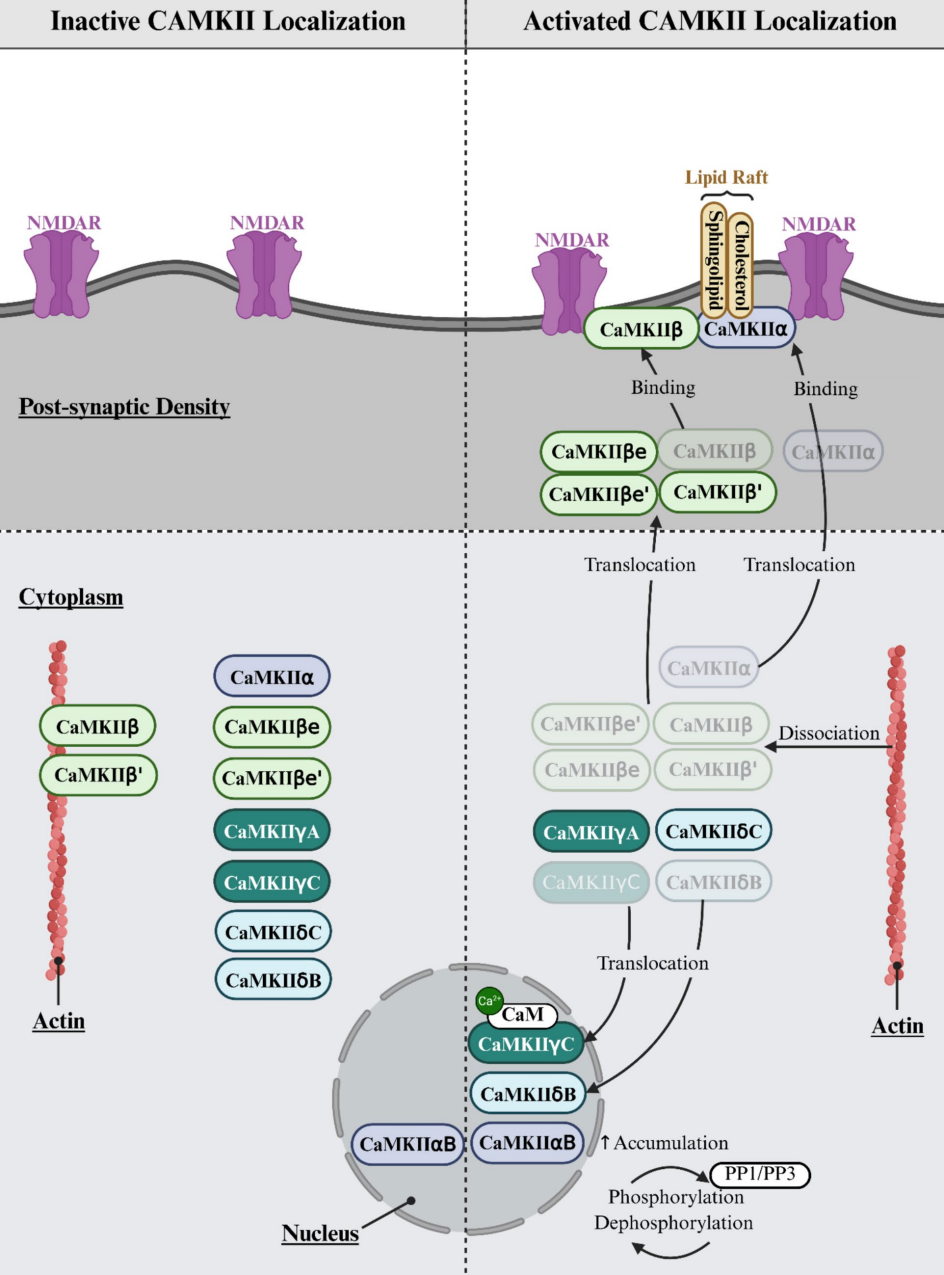
Isoform-specific localisation of CaMKII in inactive and activated states. In the inactive state, CaMKIIα, β, γ, and δ isoforms reside in the cytoplasm, with CaMKIIβ bound to actin and low nuclear CaMKIIαB/δB. Upon activation, CaMKIIα/β translocate to the postsynaptic density and bind GluN2B, while γA/γC/δC/δB translocate to the nucleus. CaMKIIγ shuttles Ca^2+^/CaM to sustain CREB phosphorylation, with nuclear CaMKII regulated by PP1/PP3. *Made with BioRender*

Isoform diversity adds further nuance. CaMKIIβ, but not CaMKIIα, binds F-actin through its N-terminal actin-binding domain [94]. F-actin binding spatially anchors CaMKII within spines, increases the likelihood of repeated Ca^2+^/CaM binding, slows deactivation by sterically hindering autoinhibitory domain reassociation, and biases Thr286 phosphorylation [18, 95] (Fig. 3). Additional scaffolds shape localisation and signalling persistence. For example, α-Actinin enhances CaMKII retention within spines and contributes to cytoskeletal reorganisation, while CASK influences non-catalytic autophosphorylation states such as Thr306/307 [16, 17, 96]. AKAP79/150 forms a nanodomain complex containing CaMKII, PKA, and PP2B, positioning the kinase near Ca^2+^ microdomains and AMPAR/NMDAR machinery. This scaffold effectively tunes phosphorylation-dephosphorylation cycles and stabilises CaMKII at potentiated synapses [97].

#### Redox and Metabolic Modifications as Non-canonical Activation Modes

A major shift in our understanding of CaMKII regulation came from recognising that autonomous activity can arise through redox and metabolite-dependent modifications within the regulatory segment. The earliest demonstration of CaMKII activation beyond Ca^2+^/CaM and Thr286 autophosphorylation came from Erickson et al. (Cell, 2008), who showed that oxidation of regulatory domain residues Met281/Met282, or closely corresponding positions across CaMKII isoforms, prevents re-engagement of the autoinhibitory segment with the catalytic cleft and generates Ca^2+^-independent activity during oxidative stress [9, 70]. Oxidation at these positions produces a stable ox-CaMKII state that persists until reversed by methionine sulfoxide reductase A (MsrA), linking kinase activity to intracellular ROS levels [9]. Coenzyme A (CoA) provides an additional metabolic mode of regulation by binding the regulatory segment and destabilising autoinhibition, enabling CaMKII activation even at basal Ca^2+^/CaM levels [11]. Other post-translational modifications including O-GlcNAcylation near Ser279 and S-nitrosylation further tune kinase responsiveness by altering regulatory-segment accessibility and ATP/substrate affinity [13, 98]. Together, these modifications reveal that CaMKII activation is governed by diverse redox and metabolite-dependent biochemical switches, beyond the traditional CaM/Thr286 pathway.

#### Integrating the Evidence: An Updated Model for CaMKII Modulation

Collectively, these findings reposition CaMKII as a highly adaptable signalling hub whose activation state arises from the combined influence of several regulatory layers rather than a single biochemical switch. Redox and metabolite-driven modifications can generate Ca^2+^ independent activity. Interactions with protein binding partners stabilise open conformations and prolong signalling after the initial Ca^2+^/CaM pulse. Higher-order structural dynamics, including kinase domain dimerisation and activation-triggered subunit exchange, propagate activation within and between holoenzymes and amplify local signals. Cytoskeletal and scaffold proteins such as F-actin, α-actinin, CASK, and AKAP79/150 anchor CaMKII within defined nanodomains and tune its access to upstream and downstream effectors. These processes operate within phase-separated and spatially restricted synaptic compartments that maintain CaMKII engagement long after Ca^2+^ transients have ended.

## Isoform Specific Localisation and Functional Specialisation

CaMKII isoforms display distinct, activity-dependent subcellular localisation patterns that underpin their specialised functions across brain regions and neuronal subtypes (Fig. 3).

### CaMKIIα

CaMKIIα is highly enriched in forebrain excitatory neurons, particularly in the hippocampus, neocortex and amygdala, where it drives LTP and memory formation [67]. CaMKIIα is mainly cytoplasmic at rest (Fig. 3), whereas the αB splice variant contains a nuclear localisation signal (NLS) and shows strong nuclear accumulation in the hypothalamus, suggesting region-specific transcriptional roles [4, 24, 63, 64].

Upon activation (Fig. 3, right panel), CaMKIIα rapidly translocates to the postsynaptic density (PSD) where it binds GluN2B, anchoring the holoenzyme within nanometres of NMDARs to optimise Ca^2+^-dependent signalling [2, 19–21, 28, 65]. Interaction with CaMKIIβ and its F-actin-binding domain (upper right) further stabilises CaMKIIα at active synapses [16]. CaMKIIα also enriches in cholesterol and sphingolipid lipid rafts (top right), promoting PSD95 assembly and PSD enlargement [99].

Nuclear targeted αB (lower right) enters the nucleus independently of Ca^2+^/CaM, but import is regulated by phosphorylation near the NLS. PP1 and calcineurin promote dephosphorylation during activity and increase nuclear entry [4, 23, 64]. Importantly, CaMKIIα protein levels at synapses can rise during plasticity through local translation of CaMKIIα mRNA [100]. Dendritic CaMKIIα transcripts are anchored near spines and translated in response to Ca^2+^ influx and neuromodulatory signalling, ensuring rapid, spatially restricted increases in CaMKIIα precisely where potentiation occurs [101].

### CaMKIIβ

CaMKIIβ is widely expressed across the brain and is dominant in the cerebellum and hypothalamus, complementing CaMKIIα-rich hippocampal and cortical regions [4, 102]. β and β′ isoforms contain an F-actin-binding domain and localise strongly to dendrites and spines (Fig. 3), while βe and βe′ lack part of this domain and show more diffuse cytoplasmic distribution [4]. Functionally, CaMKIIβ stabilises actin and spine structure at rest (Fig. 3, left panel). Upon Ca^2+^/CaM activation, it partially dissociates from actin and moves toward the PSD (Fig. 3, right panel), where it cooperates with CaMKIIα to support structural remodelling [4, 28]. CaMKIIβ can bind GluN2B, but its longer linker reduces its ability to form phase-separated CaMKII-GluN2B assemblies, limiting its contribution to LTP compared with CaMKIIα [66]. Unlike α- and some δ- and γ-variants, CaMKIIβ rarely enters the nucleus [4].

### CaMKIIγ

CaMKIIγ is expressed in brain, heart, and immune tissues, with γA and γC enriched in hippocampus and cortex [24, 67]. γ isoforms are cytoplasmic at rest but undergo activity-dependent nuclear translocation (Fig. 3), particularly γA, following Ca^2+^/CaM binding [22, 68]. Thr287 phosphorylation by CaMKIIβ stabilises Ca^2+^/CaM during nuclear transit, allowing CaMKIIγ to act as a shuttle that delivers Ca^2+^/CaM to the nucleus (lower right), sustaining CREB activation and driving gene transcription programmes that support synaptic plasticity and axon growth [12, 68, 103] 

### CaMKIIδ

CaMKIIδ is present in the brain and peripheral tissues including heart, skeletal muscle, and immune system [24]. In the CNS, it is expressed in the hippocampus and brainstem and localises to axons and presynaptic terminals during development, where it supports neurite growth and structural stability (Fig. 3, left panel) [69]. δB and δC isoforms differ in localisation. δB contains an NLS and enters the nucleus to regulate transcription (lower right), while δC remains cytoplasmic and modulates signalling and calcium handling [23, 68]. During neuronal activation, δ isoforms can translocate to the PSD and nucleus (Fig. 3, right panel), supporting synaptic and transcriptional plasticity [23, 67]. In peripheral tissues, δ isoforms regulate calcium homeostasis and stress-induced gene expression [27].

### CaMKII Localisation and Function in the Growth Cone

Despite its functional importance, the subcellular localisation and isoform-specific roles of CaMKII in growth cones remain far less studied compared to its well characterised localisation in dendritic spines and synapses. CaMKII plays a pivotal role in growth cone motility and axon guidance by linking local Ca^2+^ signals to cytoskeletal remodelling [40]. Among neuronal isoforms, CaMKIIβ is the principal isoform enriched in growth cones due to its F-actin-binding domain, which anchors holoenzymes within filopodia and lamellipodia and stabilises actin structures necessary for directional advance [18, 104]. Other isoforms, including α, γ, and δ, are also present at lower levels and may contribute indirectly via mixed holoenzymes or weaker actin interactions [48, 105]. In this compartment, CaMKII regulates actin dynamics and filopodial behaviour in ways that parallel its structural roles at synapses, but with outputs tuned to axon extension, turning, and branching [32].

Importantly, growth-cone CaMKII signalling represents a broader compartmental continuum extending along the axon and into the nucleus. In developing and mature axons, CaMKII contributes to presynaptic bouton formation, maintenance, and degeneration-related signalling pathways, linking local Ca^2+^ activity to long-range structural stability. In parallel, CaMKIIγ and CaMKIIδ isoforms can translocate to the nucleus, where they couple Ca^2+^/calmodulin signalling to transcriptional programmes that regulate neuronal growth, plasticity, and long-term stability [32, 106]. Thus, growth-cone CaMKII activity forms part of an integrated spatial signalling network that connects cytoskeletal regulation in developing neurites with axonal and nuclear functions.

Together, the four CaMKII isoforms create a versatile regulatory system that integrates spatial localisation, temporal activation, and substrate specificity to couple transient Ca^2+^ signals with lasting biochemical, structural, and transcriptional responses across synapses, axons, and the nucleus.

## Integration of Catalytic and Structural CaMKII Functions

Recent findings show that non-catalytic structural interactions of CaMKII, particularly its binding to the NMDA receptor subunit GluN2B, can be sufficient to support LTP induction under specific experimental conditions [82]. When combined with autophosphorylation at Thr286, anchoring of CaMKII to GluN2B enables LTP even when kinase activity toward downstream substrates is blocked, challenging the traditional view that substrate phosphorylation is strictly required for LTP initiation [82]. These findings highlight an essential scaffolding role for CaMKII that operates alongside, rather than replacing, its enzymatic function.

Importantly, CaMKII kinase activity remains critical for downstream synaptic processes that stabilise and consolidate plasticity, including AMPA receptor phosphorylation, cytoskeletal remodelling, and activity-dependent transcriptional regulation that promote dendritic spine enlargement and long-term maintenance of synaptic changes [52, 67, 71, 107, 108]. Phosphorylation of actin regulatory proteins such as cofilin and α-actinin, along with scaffolding proteins and receptor subunits, contributes to F-actin reorganisation and structural maturation of synapses [16, 109]. Beyond dendritic spines, CaMKII catalytic activity also regulates axon growth, cytoskeletal organisation, and long-term axonal maintenance, further demonstrating that enzymatic signalling functions operate in parallel with structural anchoring mechanisms to shape neuronal architecture [16, 110]. Together, these findings support a model in which CaMKII structural and catalytic roles are functionally distinct yet mechanistically interdependent, with both contributing essential biological functions.

### Catalytic Substrates Linking Structural Anchoring to Functional Output

**Fig. 4:**
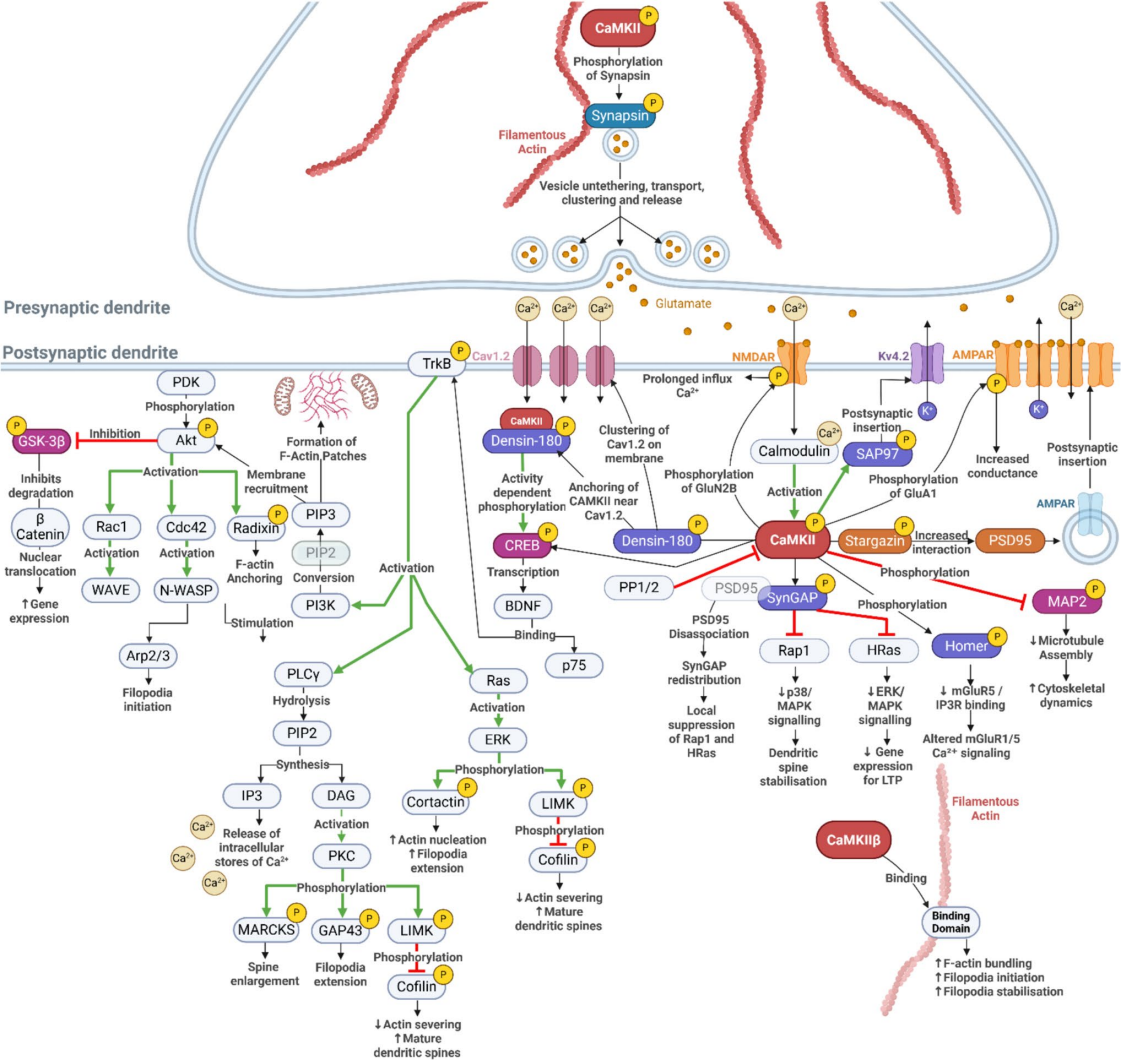
CaMKII substrates and downstream signalling pathways in pre and postsynaptic compartments. Presynaptic activation and vesicle mobilisation (blue), postsynaptic activation and receptor modulation (orange), scaffold and signalling integration (Purple), transcriptional control and structural plasticity (pink). *Made* *with* *BioRender*

The cooperative structural anchoring and catalytic activation of CaMKII ultimately influence neuronal function through phosphorylation of specific downstream substrates. These targets translate CaMKII localisation and activation state into coordinated changes in synaptic strength, cytoskeletal organisation, and activity-dependent gene expression (Fig. 4). After activation and precise compartmental localisation, CaMKII orchestrates signalling at pre- and postsynaptic sites by phosphorylating a broad array of synaptic, cytoskeletal, and transcriptional regulators, thereby converting activation state into durable functional outcomes. In the following sections, we highlight key CaMKII kinase-dependent substrates (Fig. 4).

#### Presynaptic Activation and Vesicle Mobilisation

##### Synapsin

CaMKII phosphorylates synapsin at Ser603, weakening its binding to F-actin and synaptic vesicles and mobilising the reserve pool toward the active zone [111, 112]. Under basal conditions, dephosphorylated synapsin restrains vesicles by cross-linking them to actin [113]. In CaMKIIα presynaptic knockouts, high-frequency stimulation produces excessive release and reduced depression despite reduced synapsin phosphorylation, suggesting additional CaMKII targets regulate presynaptic excitability [112]. These include N-type Ca^2+^ channels, through which CaMKII helps maintain vesicle release within a functional dynamic range [114].

#### Postsynaptic Activation and Receptor Modulation

##### N-methyl-D-aspartate Receptor (NMDAR)

NMDAR-driven Ca^2+^ influx activates CaMKII, which binds GluN2B and stabilises an open, active conformation required for autonomous activity [30, 115]. CaMKII phosphorylates GluN2B at Ser1303, reducing receptor desensitisation and thereby sustaining NMDAR-mediated Ca^2+^ currents during prolonged synaptic activity. This enhanced and prolonged Ca^2+^ influx promotes the induction and stabilisation of LTP by reinforcing CaMKII signalling at active synapses [30]. Consistent with this, loss of CaMKIIα abolishes LTP, and kinase-dead rescue only partially restores function, indicating that catalytic phosphorylation of substrates such as GluN2B is required alongside non-catalytic scaffolding roles [116].

##### Αamino-3-hydroxy-5-methyl-4-isoxazoliopropionate Receptor (AMPAR)

CaMKII phosphorylates GluA1 at Ser831, increasing channel conductance and supporting LTP-associated AMPAR insertion [71, 117, 118]. Kinase-dead CaMKII or CaMKIIα/β knockdown eliminates this phosphorylation and blocks synaptic strengthening [67, 71].

##### Stargazin

CaMKII phosphorylation of stargazin at Ser684 and Thr681 enables PSD-95 binding and immobilises AMPARs at synapses [119]. CaMKII inhibition prevents this process and blocks activity-dependent AMPAR trapping, whereas phospho-mimetic stargazin enhances synaptic AMPAR content independently of stimulation [119].

#### Scaffold and Signalling Integration

##### SynGAP

CaMKII phosphorylates SynGAP at multiple sites, shifting its Ras and Rap GAP activity to favour Ras-ERK signalling that drives AMPAR insertion and spine maturation [120, 121]. Loss of CaMKII catalytic activity prevents SynGAP phosphorylation and disrupts this signalling balance [15, 120].

##### Densin180

CaMKII binds densin-180 and phosphorylates Ser1397, stabilising PSD scaffolds and coupling Ca^2+^ influx to CREB signalling that drives activity-dependent gene transcription, including plasticity and survival programmes that support synapse strengthening and dendritic growth [78]. Blocking CaMKII activity reduces densin phosphorylation and weakens PSD structure and synaptic strength [122].

##### SAP97

CaMKII phosphorylates SAP97 at Ser39 to drive forward trafficking of SAP97 and Kv4.2 into dendrites and at Ser232 to regulate PDZ-domain interactions with NMDAR subunits [123, 124]. CaMKII inhibition prevents SAP97 and Kv4.2 surface delivery and reduces A-type currents [123, 125, 126].

##### Homer

CaMKII phosphorylates long Homer isoforms at hinge-region serines, reducing mGluR5 binding affinity and enabling activity-dependent reorganisation of mGluR-IP3R signalling complexes [127]. This remodelling permits flexible coupling of mGluR5 to downstream Ca^2+^ release pathways, supporting synaptic growth and plasticity, whereas CaMKII inhibition stabilises these complexes and constrains adaptive signalling [127].

#### Transcriptional Control and Structural Plasticity

##### CREB

CaMKII phosphorylates CREB at Ser133 to initiate transcription of neurotrophic and structural proteins including BDNF and downstream structural plasticity [85, 128, 129]. Blocking CaMKII or Ca^2+^ influx prevents Ser133 phosphorylation and BDNF induction [85].

##### MAP2

CaMKII phosphorylation of MAP2 reduces its microtubule-stabilising activity, enabling dendritic and spine remodelling during neuronal activity [130]. CaMKII inhibition prevents MAP2 phosphorylation and restricts structural plasticity [130, 131].

##### βcatenin/GSK3β

CaMKII inhibits GSK3β through PI3K–Akt signalling and can also directly phosphorylate GSK3β at Ser9 to suppress its activity. GSK3β is a constitutively active kinase that negatively regulates neuronal survival, synaptic plasticity, and cytoskeletal stability by limiting CREB-dependent transcription and destabilising microtubules. CaMKII inhibition elevates GSK3β activity and disrupts survival and plasticity pathways [43, 132].

## Regulation of CaMKII by Phosphatases in a Multistate Framework

**Fig. 5:**
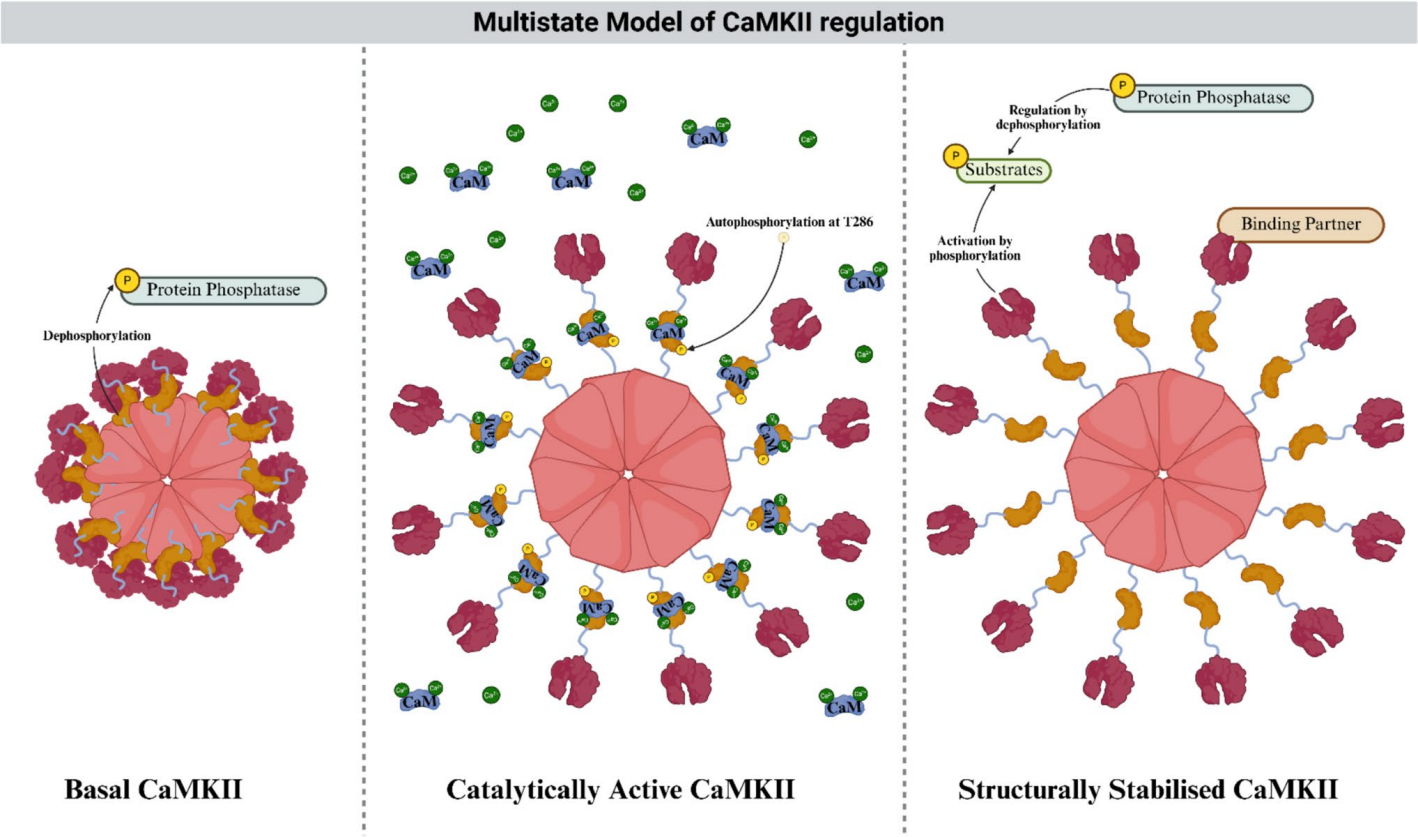
Conceptual multistate model of CaMKII regulation. Schematic illustrating three functionally distinct regulatory regimes of the CaMKII holoenzyme. Left: In the basal regime, CaMKII adopts an autoinhibited conformation with low catalytic output. Centre: Ca^2+^/calmodulin binding and Thr286 autophosphorylation promote a catalytically active, LTP-associated regime. Right: Sustained interactions with binding partners and synaptic scaffolds support a structurally stabilised regime characterised by spatial retention and protein complex formation despite reduced catalytic activity. These regimes represent a conceptual framework for organising experimental observations rather than formally established dynamical attractor states. Transitions between regimes are shaped by phosphatase activity, multivalent protein interactions, and nanoscale spatial confinement. *Made with* *BioRender*

CaMKII acts as a pivotal integrator of calcium signals in neurons, converting transient Ca^2+^ elevations into long-lasting biochemical and structural states. Classical models portrayed CaMKII regulation as a bistable kinase and phosphatase toggle, in which PP1, PP2A, and calcineurin oppose CaMKII autophosphorylation to maintain reversible cycling between high and low phosphorylation states [103]. While this framework captured essential aspects of CaMKII autonomous activity, it does not fully account for the spatial persistence and structural retention observed in multiple experimental contexts. Instead, emerging evidence supports a conceptual multistate model in which CaMKII can exist in basal, catalytically active, and structurally stabilised regimes with distinct regulatory properties (Fig. 5) [40, 77]. These regimes are inferred from experimental observations of phosphorylation state, catalytic activity, and synaptic retention rather than from formal dynamical demonstrations of discrete stable attractor states. Indeed, formal dynamical criteria for tristability, for example, experimentally demonstrated hysteresis, have not yet been established for CaMKII, and spatially heterogeneous Ca^2+^ microdomains further complicate strict classification. Accordingly, we present this framework as a unifying conceptual model that integrates biochemical, structural, and spatial observations rather than as a definitive dynamical system.

The mechanistic determinants governing transitions between these states are likely to be critically shaped by phosphatase activity. Rather than acting as simple off-switches, PP1, PP2A, and calcineurin could exert distinct yet complementary influences on CaMKII autophosphorylation kinetics, spatial localisation, isoform-specific retention, and signalling duration [77]. We propose that differential phosphatase engagement biases CaMKII toward specific basins within the multistate landscape, thereby regulating not only whether CaMKII is active, but which state it occupies, for how long, and in which subcellular compartment. PP1 is a primary regulator of transitions out of the autonomously active catalytic state [133, 134]. At the postsynaptic density, PP1 forms stable complexes with CaMKIIα and rapidly dephosphorylates Thr286 following LTP induction, resetting CaMKII, and preventing inappropriate persistence of autonomous activity [49]. By restoring CaMKII to basal phosphorylation levels, PP1 helps determine the exit from the catalytic LTP-like state [134].

PP2A provides broader regulatory control by modulating both α and β CaMKII isoforms throughout dendrites [36]. Through dephosphorylation of key regulatory sites, PP2A influences dendritic spine morphology and AMPAR and NMDAR trafficking, and regulates the spatial distribution of CaMKII within the postsynaptic density [36]. Biochemical reconstitution and imaging experiments show that PP2A directly dephosphorylates Thr286-autonomous CaMKIIα within the PSD, weakening its high-affinity interaction with NMDAR-associated complexes and returning the kinase to a basal, extrasynaptic scaffold. This phosphatase-driven dephosphorylation dynamically repositions CaMKII between distinct postsynaptic nano domains in an activity-dependent manner, rather than simply switching its catalytic state [77]. By altering CaMKII localisation and functional state, PP2A helps determine whether CaMKII remains in basal, catalytic, or structurally stabilised configurations [36, 37]. Disruption of PP2A activity perturbs this balance, leading to abnormal CaMKII retention at synapses, altered spine morphology, and synaptic dysfunction, and has been implicated in neurodevelopmental and neurodegenerative disorders linked to dysregulated CaMKII signalling [135].

Calcineurin (PP2B) is activated by sustained Ca^2+^ elevations and regulates actin cytoskeleton remodelling through dephosphorylation of CaMKII, influencing the structural state of CaMKII and the morphology of dendritic spines [106, 136]. Calcineurin also promotes nuclear translocation of CaMKIIγ, enabling CREB-dependent transcription required for long-term plasticity and memory formation [137].

In addition to restraining CaMKII itself, protein phosphatases also counterbalance CaMKII signalling by dephosphorylating multiple downstream substrates, including GluA1, GluN2B, Tiam1, and SynGAP (Fig. 5) [120, 138–140]. By actively reversing these substrate level phosphorylation events, phosphatases provide a crucial checkpoint that limits CaMKII-driven signalling arising from non-canonical activation modes. This feedback prevents runaway CaMKII activity that would otherwise destabilise synaptic function and become toxic to neurons.

Together, PP1, PP2A, and calcineurin form a coordinated phosphatase network that defines the stability, timing, and localisation of CaMKII activity across its three states. Rather than simply terminating kinase activity, these phosphatases enforce the multistate CaMKII signalling landscape, ensuring that CaMKII-driven plasticity remains reversible, compartmentalised, and tightly regulated.

## The Overlooked Role of CaMKII Isoforms in Axon Growth, Maintenance, and Degeneration

Although CaMKII is traditionally viewed as a synaptic plasticity kinase, a growing body of evidence shows that specific CaMKII isoforms exert powerful control over axonal structure, growth, and susceptibility to degeneration. CaMKIIα and CaMKIIβ, the two predominant neuronal isoforms, have distinct yet complementary roles in axon development and maintenance. CaMKIIβ, which contains a high-affinity F-actin-binding domain, is a key regulator of growth cone actin organisation. It supports filopodial extension, branching, and turning responses, and genetic or pharmacological inhibition of CaMKIIβ disrupts axon elongation in cortical and sensory neurons [33, 104, 141]. CaMKIIα also contributes to axon outgrowth by regulating microtubule-associated proteins and coordinating calcium-dependent growth cone motility [142]. At presynaptic boutons, CaMKIIα is required for activity-dependent bouton formation and long-term enlargement, linking electrical activity to structural stabilisation of axon terminals [110].

CaMKII also intersects with scaffold-mediated control of the cytoskeleton. Phosphorylation of AKAP79 or AKAP150 by CaMKII reduces its binding to actin in vitro [97]. This mechanism has been primarily investigated in dendritic spines during long-term depression where AKAP79 or AKAP150 dispersal is required for structural remodelling, but it highlights a broader principle in which CaMKII modulates both actin networks and scaffold proteins that organise signalling complexes.

Emerging evidence now connects CaMKII signalling to the SARM1 programmed axon degeneration pathway. Ding et al. (2022) demonstrated that CaMKIIα activation suppresses SARM1-driven degeneration in models of mitochondrial stress through modulation of an upstream CaMKII to ASK1 to p38 MAPK axis [143]. This identifies CaMKIIα as a pro-survival kinase that counterbalances metabolic and stress-related triggers of SARM1 activation. It also reveals that neuronal activity and calcium influx, historically interpreted only as synaptic signals, can directly shape axonal fate by modulating NAD-sensitive pro-degenerative pathways.

Together, these findings establish that CaMKIIα and CaMKIIβ are not confined to synaptic plasticity but are central regulators of axon growth, long-term structural maintenance, and vulnerability to programmed destruction. Incorporating CaMKII isoform-specific biology into models of axon degeneration may uncover new therapeutic strategies that preserve axons while maintaining the essential activity-dependent survival signals required for neuronal health.

## Neurological and Neurodegenerative Disorders Linked to CaMKII Dysregulation

Dysregulation of CaMKII signalling is implicated across a range of neurodevelopmental, neuropsychiatric, and neurodegenerative disorders, summarised in Table 1.

**Table 1:** Summary of neurological and neurodegenerative disorders associated with CaMKII Dysregulation

DISEASE/disorder	CaMKII isoform(s)	Type of CAMKII-related dysregulation	Brain region(s)	Key downstream effects	Clinical/behavioural phenotype
Autism spectrum disorder	α, β; CaMKIV	Missense mutations leading to ↓ Thr286 autophosphorylation, mislocalisation and disrupted CaMKIIβ-actin coupling	Cortical and hippocampal circuits	↓ AMPA/NMDA phosphorylation, impaired spine stabilisation, altered excitatory/inhibitory balance	Hyperactivity, social deficits, repetitive behaviours and intellectual disability in some cases
Schizophrenia	α, β	↓ CaMKIIα autophosphorylation and NMDA coupling, altered CaMKIIβ expression	Prefrontal cortex, hippocampus	Impaired LTP-like plasticity, abnormal spine density/morphology	Cognitive deficits and impaired working memory
Epilepsy	α, β	Hyperphosphorylation and relocalisation, regionally divergent pCaMKII levels, excessive modulation of persistent Na^+^ currents	Hippocampus (↓ pCaMKII) and cortex (↑ pCaMKII)	Hyperexcitability, calcium overload/excitotoxic stress and seizure-linked kinase cycling	Recurrent seizures and neuronal injury in severe cases
Alzheimer’s disease	α	Dysregulated Thr286, mislocalisation of pCaMKIIα from spines to soma, association with Aβ	Hippocampal CA1, cortical regions	Impaired synaptic plasticity, cytoskeletal defects via MAP2, Aβ-driven suppression of activity	Memory decline and synapse loss
Parkinson’s disease	α	Oxidative CaMKII activation and chronic L-DOPA alter NMDAR trafficking	Striatum and substantia nigra circuits	↑ GluN2B surface retention, maladaptive plasticity, excitotoxic stress, D2 receptor signalling changes	Motor symptoms, L-DOPA-induced dyskinesia
Huntington’s disease	α; CaMKIV	↓ CaMKII expression and phosphorylation, aberrant oxidative activation, disrupted Ca^2+^/CaM cascades, interaction with TG2 pathway	Striatum, cortex	Impaired CREB/BDNF signalling, mitochondrial and synaptic dysfunction, kinase network imbalance (ERK2, CDK5)	Motor abnormalities, cognitive decline
Glaucoma and retinal ganglion cell degeneration	α, β (retinal)	↓ autophosphorylation ↓ CaMKII to CREB and BDNF to TrkB signalling	RGC somas and optic nerve	↓ CaMKII and CREB activity → RGC soma and axon loss	Progressive visual field loss and blindness

In autism spectrum disorder, mutations in CaMKIIα and CaMKIIβ impair Thr286 autophosphorylation, weaken spine targeting, and reduce modulation of glutamate receptors, while disrupted CaMKIIβ-actin coupling and altered phosphatase regulation destabilise spine nanodomains [67, 144–149]. Schizophrenia features reduced CaMKIIα/β expression and impaired autophosphorylation, compromising NMDAR-dependent plasticity and PSD organisation [57, 150–152]. Epilepsy presents region-specific abnormalities in CaMKII phosphorylation and localisation that alter intrinsic excitability and network dynamics [67, 153–156]. Neurodegenerative disorders show similar convergence on CaMKII misregulation. In Alzheimer’s disease, dysregulated Thr286 phosphorylation, CaMKII mislocalisation, and pathological interactions with amyloid-β and tau impair synaptic plasticity [157–159]. In Parkinson’s disease, oxidative generation of autonomous CaMKII drives maladaptive GluN2B signalling [106, 160–162]. Huntington’s disease involves reduced CaMKII expression and Thr286 phosphorylation, disrupting CREB-BDNF pathways [67, 163, 164]. Conversely, retinal and optic nerve degeneration reflects a harmful loss of CaMKII activity that compromises neuronal survival [165, 166]. Together, these disorders highlight CaMKII misregulation as a shared pathogenic mechanism across brain disease, where disturbances in CaMKII’s activation, localisation, or integration into synaptic signalling pathways translate into impaired plasticity, altered network function, and progressive loss of neuronal stability.

## Challenges and Opportunities in Therapeutic Targeting of CAMKII

Despite strong preclinical evidence that carefully tuning CaMKII activity can protect neurons, correct abnormal patterns of substrate phosphorylation, and improve synaptic and cognitive function [167–169], clinical translation is yet to be achieved. Effective therapeutics will likely need to target both directions of dysregulation, from suppressing pathological overactivation of CaMKII when its signalling is excessive and enhancing CaMKII-dependent phosphorylation of key substrates when activity is insufficient to support normal plasticity and neuronal survival.

### Isoform Diversity and Selective Targeting

CaMKII is a challenging therapeutic target because it exists as multiple isoforms and splice variants that occupy distinct structural and catalytic states across different subcellular compartments. CaMKIIα and CaMKIIβ dominate in forebrain spines where they regulate LTP and structural plasticity, whereas CaMKIIγ and CaMKIIδ contribute to nuclear signalling, calcium handling, and peripheral organ function [18–24]. Even within a single neuron, individual isoforms participate in multiple functional pools, such as GluN2B-bound CaMKIIα at the PSD, F-actin tethered CaMKIIβ in growth cones, and nuclear CaMKIIγ that sustains CREB activation [8, 170, 171]. As a result, broad ATP competitive inhibitors or global activators risk suppressing essential synaptic plasticity, disrupting survival pathways, and interfering with peripheral CaMKII functions.

Disease processes often hijack specific activation mechanisms. Oxidised CaMKII species are characteristic of cardiovascular and Parkinsonian pathology, while excessive CaMKII-GluN2B binding and phase separation contribute to excitotoxicity and maladaptive striatal plasticity [8, 172]. This suggests that therapies should target the disease-relevant activation mode rather than the catalytic domain. Potential strategies include preventing pathological oxidation of Met281 and Met282, normalising CoA regulated activation or disrupting excessive CaMKII-GluN2B condensates [9, 11–17]. Phosphatases further shape the CaMKII landscape. PP1, PP2A, and calcineurin determine how long CaMKII remains in basal, catalytic, or structurally stabilised states [37, 72, 77, 103, 133–135, 173, 174]. When CaMKII becomes pathologically persistent, adjusting phosphatase activity may be more effective than direct kinase inhibition [10]. Conversely, when CaMKII activity is too low to support CREB or BDNF signalling, reducing opposing phosphatase tone may help restore appropriate activation [2]. These approaches will require spatial precision given the broad roles of these phosphatases. sFuture therapeutic progress will likely rely on isoform-selective modulators, agents that correct pathological protein interactions or redox and metabolic modifications, and strategies that tune phosphatase feedback rather than simply blocking CaMKII activity.

## Conclusion

CaMKII should be understood as a dynamic signalling switch whose influence depends not on simple increases or decreases in kinase activity but on the precise state, location, and duration of its activation. Its structural and catalytic roles, shaped by isoform diversity, partner interactions, and phosphatase control, determine how neurons adapt, stabilise, or fail under changing conditions. Disorders emerge when these regulatory relationships are pushed out of balance, not when CaMKII is merely “too active” or “not active enough”. The challenge moving forward is to develop interventions that restore appropriate state control without disrupting the flexibility that allows CaMKII to support healthy neuroplasticity.

## Data Availability

No datasets were generated or analysed during the current study.
